# Diet, Maternal Nutrition, and Reproductive Health—From Preconception to Postpartum and Beyond

**DOI:** 10.3390/nu18030412

**Published:** 2026-01-26

**Authors:** Angela Vinturache

**Affiliations:** 1 Department of Obstetrics and Gynecology, University of Alberta, Edmonton, AB T6G 2R3, Canada; angela.vinturache@gmail.com; 2 Department of Neuroscience, University of Lethbridge, Lethbridge, AB T1K 3M4, Canada

## 1. Introduction

Nutrition is a foundational determinant of reproductive health across the life course—from preconception and fertility through pregnancy, lactation, and menopause. This *Nutrients* Special Issue “**Diet, Maternal Nutrition, and Reproductive Health**” brings together 12 papers that collectively advance three priorities for the field: (1) improving how we measure diet, nutrient status, and nutrition knowledge in real-world settings; (2) clarifying how the maternal diet and metabolic context shape micronutrient transfer and offspring outcomes; and (3) refining the benefit–risk framing around supplementation, particularly for nutrients that are widely recommended during pregnancy [1,2,3,4,5,6,7,8,9,10,11,12].

A key strength of this Special Issue lies in the methodological rigor of the included studies, which apply robust exposure assessment approaches—ranging from objective dietary measurement to biologic matrices that more closely capture true maternal–fetal nutrient exposure—while also explicitly situating nutritional risk and nutrient status within socio-demographic contexts that shape access, adequacy, and inequities [1,2,3,4,5,6,7].

## 2. Original Research in Humans: Moving from “Intake” to Biologically Relevant Exposure and Equity-Aware Interpretation

### 2.1. Biomonitoring Fetal Micronutrient Exposure Using Meconium

Mimica and colleagues quantify iron (Fe), zinc (Zn), copper (Cu), and manganese (Mn) in neonatal meconium and relate these concentrations to maternal diet, lifestyle, and newborn anthropometrics [1]. Meconium is an underused but conceptually powerful matrix: it integrates fetal exposures over time rather than reflecting only a narrow sampling window. The authors report substantial interindividual variability and a particularly strong positive correlation between Fe and Cu, which is consistent with shared transport or regulatory pathways [1]. Their findings also align fetal trace element status with the maternal metabolic context—pre-pregnancy BMI was associated with lower meconium Fe levels, while BMI at delivery was related to Zn—supporting the broader literature that suggests that maternal adiposity can influence placental nutrient handling [1]. Importantly, specific dietary patterns tracked with meconium metals (e.g., fruit intake with Cu; vegetables with lower Fe levels; tea with higher Zn levels), while supplement use exhibited no clear association, underscoring how whole-diet exposures and absorption modifiers may matter as much as supplementation in some settings [1]. Clinically, the observed links between maternal meat intake and a greater birth size reinforce longstanding evidence on the importance of adequate protein and bioavailable micronutrients for fetal growth, while also raising a mechanistic question: what dietary patterns promote optimal micronutrient transfer without increasing risks associated with excessive gestational weight gain? [1].

### 2.2. Gold-Standard Dietary Assessment to Identify “Quiet” Gaps in Adequacy

Osei et al. address a perennial challenge in maternal nutrition research: dietary misclassification driven by self-reports. Using researcher-conducted weighed food records in rural and urban Ghanaian mothers, they provide unusually robust estimates of energy and nutrient intake [2]. This methodological choice is a key contribution, particularly in contexts where household measures and recall-based methods can systematically distort intake patterns. While most participants met recommended intake levels for several nutrients, folate inadequacy remained common—affecting roughly half of mothers in both rural and urban settings—despite macronutrient distributions falling within recommended ranges [2]. This paper advances the field by demonstrating that even when overall diets appear adequate, specific micronutrients can remain limiting; this is precisely the scenario in which targeted fortification, supplementation strategies, and food-based interventions should be evaluated for effectiveness and equity [2].

### 2.3. Harmonizing Preconception Diet Quality Across Cohorts: A Prerequisite for Actionable Evidence

The PrePARED Consortium paper by Ji et al. tackles a different but equally limiting barrier: heterogeneity in dietary instruments across studies [3]. By harmonizing data from seven preconception cohorts (over 56,000 participants) using the FIGO nutrition checklist, the authors demonstrate that it is uncommon for women to meet all recommended criteria—even in populations not typically characterized by overt undernutrition [3]. Particularly striking is the consistency of gaps around processed foods and snacks, alongside variable adherence to fruit, vegetable, whole grain, dairy, and fish recommendations across cohorts [3]. The novelty here is not simply the scale but the pragmatic harmonization framework: this work enables future cross-cohort analyses that link preconception diet quality to fertility, pregnancy outcomes, and longer-term maternal cardiometabolic risk, while reducing the “apples to oranges” problem that has impacted progress in preconception nutrition science [3].

### 2.4. Perinatal Calcium and Vitamin D in Japan: Low Intake, Low Status, and the Complexity of Short-Term Skeletal Readouts

Ichikawa and colleagues evaluate calcium and vitamin D intake in postpartum Japanese women and relate intake to serum markers and bone assessments via quantitative ultrasonography (QUS) [4]. Their data document very low calcium intakes relative to national recommendations (with most participants below the RDI) and a meaningful proportion below the vitamin D RDI, accompanied by low 25(OH)D levels [4]. A particularly informative aspect here is the dissociation between intake and short-term skeletal markers/QUS indices—an expected but important reminder that calcium homeostasis is tightly regulated and that timing (third day postpartum) may obscure physiologic relationships relevant over longer intervals [4]. From a policy and counseling standpoint, their discussion is pragmatic: current Japanese obstetric guidance does not specify vitamin D intake requirements, and because fish is a primary dietary vitamin D source, guidance to limit fish for mercury avoidance may inadvertently reduce vitamin D intake—supporting a rationale for clearer guidance and/or structured supplementation and fortification strategies [4].

### 2.5. Twin Gestations and Vitamin D: When Nutrient Status May Not Be the Limiting Factor for Size

Wierzejska et al. address a clear knowledge gap: vitamin D status and fetal growth relationships in twin pregnancies, where nutrient demands and obstetric determinants differ from singleton gestations [5]. In this cohort of 50 women (100 newborns), neither maternal nor cord blood 25(OH)D concentrations were associated with newborn anthropometrics; instead, chorionicity and maternal height were the dominant correlates of birth size [5]. This “null” association is not a failure of their hypothesis—it is a meaningful refinement of clinical reasoning. When the prevalence of vitamin D deficiencies is low in the sampled population, growth variation may be more strongly driven by placentation biology and constitutional factors than by vitamin D status [5]. This study therefore helps calibrate expectations about where vitamin D screening and intervention may have the greatest marginal yield, particularly in multiple gestations [5].

### 2.6. Nutrition Knowledge as an Intervention Target: Validating a Practical Tool

Callegaro and colleagues contribute an implementation-oriented advancement by developing and validating the Italian Pregnancy Nutrition Knowledge Questionnaire (ItPreNKQ) [6]. With acceptable internal consistency and strong test–retest reliability, this tool provides a standardized protocol to identify knowledge gaps and evaluate educational interventions in prenatal care or public health settings [6]. The key “new” element here is not the observation that gaps exist but the availability of a validated, context-specific instrument aligned with national guidelines—an essential step if nutrition education is to be measurable, scalable, and comparable across programs [6].

### 2.7. Social Patterning of Trace Elements in U.S. Women: Equity Is a Micronutrient Issue

Peng et al. use NHANES (1999–2018) to examine socio-demographic determinants of serum iron, copper, zinc, and selenium levels among U.S. women of reproductive age [7]. Their analysis demonstrates that lower iron concentrations and higher risks of insufficiency cluster among older women and those who are Black, lower-income, or with lower education; patterns for zinc and selenium similarly disadvantage Black women, while copper concentrations trend higher in Black women [7]. This work’s contribution is twofold: it updates population-level evidence across long time horizons, and it reinforces that nutritional vulnerability is not evenly distributed. For reproductive health practice and policy, this supports the prioritization of replenishment planning, food access interventions, and culturally responsive counseling strategies for groups at the highest risk of insufficiency [7].

## 3. Animal and Mechanistic Studies: Clarifying Pathways, Dose–Response Concerns, and Life-Stage Specificity

The two animal studies in this Special Issue provide mechanistic depth and raise clinically relevant questions about doses, timing, and life-stage contexts—issues that are difficult to resolve through observational human studies alone [8,9].

### 3.1. High Maternal Folic Acid Supplementation and Offspring Neurodevelopment: Moving Beyond Outcomes to Cell-Type Vulnerability

Xu and colleagues apply spatial transcriptomics and single-nucleus multi-omics to a mouse model of high maternal folic acid supplementation during the periconceptional period [8]. While folic acid supplementation is a cornerstone of neural tube defect prevention, this study addresses the increasingly discussed question of whether an excessive intake may have unintended effects. The authors identify altered gene pathways linked to neurogenesis and myelination across multiple brain regions, with signals related to learning and memory in thalamic and ventricular regions [8]. Importantly, they pinpoint maturing excitatory neurons in the dentate gyrus as particularly vulnerable, revealing changes in gene expression and chromatin accessibility in ribosomal biogenesis pathways relevant to synaptic formation [8]. The novelty here is the resolution: this is not simply a “high folate changes the brain” situation but rather a map of where and in which cell populations transcriptional and epigenetic changes occur. In reproductive nutrition, this reinforces the need for nuanced dose–response research that preserves the well-established benefits of folic acid at recommended doses while carefully interrogating higher exposure levels and contextual factors (fortification, supplement stacking, and genetic variability in folate metabolism) [8].

### 3.2. Vitamin E, Exercise, and the Ovariectomy Model: Nutrition in Reproductive Aging and Menopause

Jin et al. examine vitamin E intake and voluntary wheel running in ovariectomized mice, a commonly used model for estrogen deficiency and aspects of metabolic dysfunction relevant to menopause [9]. A key finding is that vitamin E improved skeletal muscle mitochondrial function and increased PGC1-a and AMPK-related signaling, while reducing markers of oxidative stress—effects observed even when whole-body phenotypes (fat mass, energy expenditure, and glucose tolerance) did not markedly improve with combined interventions [9]. This distinction matters: reproductive nutrition is not limited to pregnancy, and interventions may yield tissue-specific benefits that precede or exceed changes in conventional metabolic endpoints. This study strengthens the rationale for investigating antioxidant–exercise interactions in women during reproductive aging, while appropriately acknowledging that translation requires human trials [9].

## 4. Reviews and Syntheses: From Fertility Optimization to Postpartum Mental Health and Infant Development

Beyond original research, this Special Issue includes three reviews that consolidate evidence across the reproductive continuum and highlight priority areas for clinical translation [10,11,12].

Donato et al. synthesize evidence that demonstrates that a preconception lifestyle intervention is not an “adjunct” but a core clinical strategy for fertility care, particularly because nutrition, adiposity, and physical activity operate through metabolic and endocrine pathways that directly influence ovulatory function, implantation biology, and assisted reproduction outcomes [10]. This review emphasizes practical, modifiable targets—weight optimization (even modest loss), dietary pattern quality (including adherence to Mediterranean-style approaches), and calibrated physical activity—while also acknowledging the biologic “dose–response” reality that moderate activity appears beneficial, whereas very high levels may impair ovulation in some women [10]. It also highlights emerging mechanistic work linking nutritional exposures (including anti-inflammatory nutrients such as n-3 PUFAs) to endometrial gene expression and inflammatory signaling, reinforcing the plausibility of nutrition-driven improvements in reproductive competence beyond weight loss alone [10].

Purkiewicz et al. extend this Special Issue beyond pregnancy outcomes to the mother–infant dyad, reviewing breastfeeding as a biologically active, developmentally timed exposure rather than solely an infant feeding choice [11]. Their review integrates evidence across neurodevelopment (including components relevant to cognitive development), immune maturation, and sleep regulation, while also addressing the maternal dimension—such as emotional responses and psychosocial stressors—and the ways in which stress physiology (e.g., cortisol-mediated hormonal disruption) can influence lactation initiation and maintenance [11]. By considering the realities of exclusive breastfeeding challenges and weaning transitions, this paper usefully reframes “support for breastfeeding” as a clinical and systems issue—requiring anticipatory guidance, mental health awareness, and practical lactation support—rather than a purely educational intervention [11].

Finally, Centeno et al. provide a timely and methodologically structured update (a systematic review with meta-analysis) on serum 25(OH)D and perinatal mental health, an area where clinical interest has outpaced evidence quality [12]. Across pooled analyses, they report lower 25(OH)D concentrations in women with antenatal depressive symptoms and similarly lower concentrations in postpartum depression, while noting that effect estimates vary by the screening instrument used and that overall certainty is often low to very low due to heterogeneity and study limitations [12]. This review therefore strengthens the rationale for integrating nutritional status into perinatal mental health research and risk stratification, while simultaneously underscoring the need for better-designed longitudinal studies and trials that account for timing across pregnancy, baseline status, confounding, and clinically meaningful thresholds for screening and intervention [12].

## 5. Cross-Cutting Implications and Future Directions

Taken together, these papers point to several actionable directions for research and practice:

1. **Strengthen measurement and exposure classification:** Future work should deliberately combine complementary methods that reduce different sources of bias—such as gold-standard dietary assessments (e.g., weighed food records), harmonized preconception diet quality metrics (e.g., FIGO-based cross-cohort scoring), validated nutrition knowledge tools, and biologic matrices that more closely reflect integrated exposure (e.g., meconium for fetal micronutrient exposure). Such triangulation will improve causal inference and enhance comparability across settings and populations [1,2,3,6].
2. **Design for nutrition equity and implementation reach:** “Equity” here refers to systematic differences in nutritional status and micronutrient adequacy driven by socio-demographic determinants—including income, education, race/ethnicity, geography (rural/urban), and differential access to nutrient-dense foods and preventive care. Evidence in this Issue reveals that trace element insufficiency and lower nutrient concentrations cluster in socially disadvantaged groups and that meaningful inadequacies (e.g., folate) can persist across settings. Research and public health programming should therefore (a) include adequate representations of higher-risk groups; (b) evaluate whether interventions reduce—not widen—gaps; and (c) embed delivery strategies that address access barriers (affordable nutrient-dense foods, culturally tailored counseling, fortification coverage, and targeted supplementation pathways) [2,7].
3. **Refine supplementation policy using a dose–timing–baseline framework:** Folate and vitamin D remain central to preconception and pregnancy guidance, but the field increasingly requires greater precision regarding doses, timing, baseline status, and cumulative exposure (e.g., food fortification plus prenatal supplements). This Special Issue highlights both the persistent challenge of inadequacy (e.g., folate shortfalls in dietary intake) and the need to interrogate higher-exposure biology (e.g., high folic acid in animal models), supporting future studies that define optimal thresholds and identify subgroups most likely to benefit—or potentially be harmed—based on baseline biomarkers and co-exposures [2,4,5,8,12].
4. **Translate animal and mechanistic studies into human-relevant questions:** The animal studies in this Special Issue provide mechanistic signals (e.g., neuronal cell-type vulnerability under high folic acid exposure; skeletal muscle mitochondrial adaptations with antioxidant intake after ovariectomy) that should be leveraged to design human research that is both feasible and clinically interpretable. Priorities include the following: (a) aligning animal exposure levels to human-relevant intake ranges and real-world patterns (including supplement “stacking” and fortification); (b) identifying biomarkers and intermediate phenotypes (transcriptomic/epigenetic signatures, oxidative stress markers, mitochondrial function proxies) that can be measured in pregnancy or postpartum cohorts; (c) testing time-sensitive windows (periconception vs. later gestation; early vs. late menopausal transition); and (d) moving from observational associations to pragmatic trials where appropriate (e.g., biomarker-stratified supplementation or lifestyle interventions) [8,9].
5. **Expand the reproductive lens and outcomes beyond birth size:** Nutrition research should continue to address fertility, pregnancy, postpartum, and menopause as a connected continuum and evaluate outcomes beyond birthweight—such as maternal functional outcomes (fatigue, muscle function), metabolic health, neurodevelopmental trajectories, breastfeeding-related well-being, and perinatal mental health. This broader framing better reflects patient priorities and may identify earlier, modifiable predictors of long-term maternal and offspring health [9,10,11,12].

In summary, this Special Issue, “**Diet, Maternal Nutrition, and Reproductive Health**”, demonstrates that progress in reproductive nutrition depends on better exposure assessment, mechanistic clarity, and implementation-ready tools—paired with an explicit commitment to addressing social determinants of nutritional risk [1,2,3,4,5,6,7,8,9,10,11,12].

We sincerely thank the authors for their rigorous and thoughtful contributions to this Special Issue. Collectively, these papers expand the evidence base in meaningful ways—introducing new data, refined methods, and important mechanistic insights to questions that matter across the reproductive life course. Moving forward, the most impactful progress will result from integrative, cross-disciplinary efforts that link stronger exposure assessments and biomarker-informed precision with implementation-ready strategies explicitly designed to reduce inequities. By aligning methodological rigor with biological plausibility and real-world delivery, the field will be better positioned to translate emerging knowledge into practical guidance and services that improve outcomes for women and their families.

## Data Availability

Not applicable.
